# Dietary Cholesterol, Serum Lipids, and Heart Disease: Are Eggs Working for or Against You?

**DOI:** 10.3390/nu10040426

**Published:** 2018-03-29

**Authors:** Christopher N. Blesso, Maria Luz Fernandez

**Affiliations:** Department of Nutritional Sciences, University of Connecticut, Storrs, CT 06269, USA; maria-luz.fernandez@uconn.edu

**Keywords:** dietary cholesterol, eggs, heart disease, lipids, HDL, LDL, phospholipids

## Abstract

The relationship between blood cholesterol and heart disease is well-established, with the lowering of serum low-density lipoprotein (LDL)-cholesterol being the primary target of preventive therapy. Furthermore, epidemiological studies report lower risk for heart disease with higher concentrations of high-density lipoprotein (HDL)-cholesterol. There has also been considerable interest in studying the relationship between dietary cholesterol intake and heart disease risk. Eggs are one of the richest sources of cholesterol in the diet. However, large-scale epidemiological studies have found only tenuous associations between the intake of eggs and cardiovascular disease risk. Well-controlled, clinical studies show the impact of dietary cholesterol challenges via egg intake on serum lipids is highly variable, with the majority of individuals (~2/3 of the population) having only minimal responses, while those with a significant response increase both LDL and HDL-cholesterol, typically with a maintenance of the LDL/HDL cholesterol ratio. Recent drug trials targeting HDL-cholesterol have been unsuccessful in reducing cardiovascular events, and thus it is unclear if raising HDL-cholesterol with chronic egg intake is beneficial. Other important changes with egg intake include potentially favorable effects on lipoprotein particle profiles and enhancing HDL function. Overall, the increased HDL-cholesterol commonly observed with dietary cholesterol feeding in humans appears to also coincide with improvements in other markers of HDL function. However, more investigation into the effects of dietary cholesterol on HDL functionality in humans is warranted. There are other factors found in eggs that may influence risk for heart disease by reducing serum lipids, such as phospholipids, and these may also modify the response to dietary cholesterol found in eggs. In this review, we discuss how eggs and dietary cholesterol affect serum cholesterol concentrations, as well as more advanced lipoprotein measures, such as lipoprotein particle profiles and HDL metabolism.

## 1. Introduction

Cardiovascular disease (CVD) contributes to more than 17 million deaths per year globally, which accounts for nearly half of all deaths from non-communicable diseases [1]. Cardiovascular disease is primarily caused by atherosclerosis, a chronic inflammatory disease of the arteries in which the deposition of cholesterol and fibrous materials in artery walls forms a plaque or lesion [2]. Key risk factors that associate with the number of atherosclerotic CVD events include the total concentration of cholesterol found in the blood, as well as the cholesterol found in individual lipoprotein subclasses [3]. Serum concentrations of low-density lipoprotein -cholesterol (LDL-C) and high-density lipoprotein -cholesterol (HDL-C) have opposite effects on CVD risk, consistent with the role of LDL particles in the promotion of, and HDL particles in the protection against, atherosclerosis [4,5].

## 2. Cholesterol, Eggs, and Heart Disease

### 2.1. Relationship between Dietary Cholesterol and/or Egg Intake on Risk for CVD in Observational Studies

The Framingham Heart Study was one of the first studies to show a relationship between serum cholesterol and heart disease, and it was hypothesized that dietary cholesterol was a modifier of heart disease through effects on serum lipids, even though no such association was observed at the time [6,7]. This hypothesis was consistent with evidence from animal studies, such as the seminal work by Nikolai N. Anichkov in rabbits in 1913, showing large doses of cholesterol in the diet markedly induced atherosclerotic plaques in aortas [8]. Even with these early studies, it was clear that there were species-specific differences in the atherosclerotic response to large doses of dietary cholesterol, with rats being markedly more resistant than rabbits and guinea pigs [8]. The average intake of dietary cholesterol in U.S. adults is typically between 200–350 mg/day, depending on gender and age group [9]. Eggs are a major source of dietary cholesterol in the typical Western diet; one large egg yolk contains approximately 200 mg of cholesterol. The consumption of eggs and egg products contributes about a quarter of the daily cholesterol intake in the U.S. in both children and adults [10,11]. Saturated fat is known to strongly increase serum cholesterol, and eggs, which are relatively low in saturated fat, only contribute about 2.5% of total saturated fatty acid intake among U.S. adults [11].

Early observational studies demonstrated a link between dietary cholesterol and risk for CVD [12,13]; however, these initial studies failed to account for many confounding variables that may limit their findings, such as other dietary and lifestyle factors. More recent epidemiological studies typically show a lack of association between dietary cholesterol and/or egg intake and CVD risk in the general population [14,15,16]. However, there does appear to be a more consistent relationship between egg intake and CVD in diabetics [14,15], however, this is not always found [17]. Interestingly, this risk in diabetics may be related to the phosphatidylcholine content of eggs [18], and not the cholesterol since dietary cholesterol is shown to be more poorly absorbed in obese and insulin-resistant populations compared to lean individuals [19,20,21]. Phosphatidylcholine intake has been linked to the gut microbial-dependent generation of trimethylamine *N*-oxide (TMAO), a metabolite shown to promote atherosclerosis in hyperlipidemic mouse models and associated with CVD risk in human cohort studies [22,23]. However, the consumption of 2–3 eggs per day was not associated with increases in fasting TMAO concentrations in healthy, young adults [24,25], while postprandial TMAO concentrations in the plasma of healthy men were found to be markedly lower after egg intake than fish intake, a direct source of dietary TMAO [26]. The impact of egg phospholipids on CVD and TMAO concentrations in humans is likely complex and requires further research [27,28]. Berger et al. [29] conducted a systematic review and meta-analysis of 17 cohort studies examining the relationship between dietary cholesterol and CVD. Dietary cholesterol intake was not found to be significantly associated with either heart disease, ischemic stroke, or hemorrhagic stroke.

### 2.2. Serum Cholesterol Responses and Adaptations to Cholesterol Intake

Early dietary recommendations assumed that increasing dietary cholesterol intake would lead to an increase in cholesterol in the blood, which over several decades would promote the development of heart disease. However, this is an oversimplification since the serum cholesterol response to dietary cholesterol is much more complicated. Humans can produce cholesterol endogenously and most of the cholesterol in the body comes from biosynthesis [30,31]. Only about 25% of serum cholesterol in humans is derived from the diet while the rest is derived biosynthesis. The average 70 kg adult synthesizes about 850 mg cholesterol/day. If this individual was to consume 400 mg/day of dietary cholesterol and absorb 60% [32], that amounts to only 22% of cholesterol handled in the body coming from the diet (240 mg from the diet out of a total of 1090 mg); furthermore, these numbers are skewed even more towards cholesterol biosynthesis in overweight and obese people [19,20,21]. Cholesterol balance is affected by synthesis rates of cholesterol and bile acids, as well as their excretion from the body. Sterol balance studies have shown feedback inhibition of cholesterol biosynthesis and increased excretion of bile acids with high cholesterol diets [33]. Cellular cholesterol biosynthesis is tightly regulated by a transcriptional program coordinated by sterol regulatory element-binding protein-2 (SREBP-2) [34]. SREBP-2 transcriptional activity increases when cellular cholesterol is reduced to upregulate the expression of genes encoding proteins involved in cholesterol biosynthesis, such as hydroxymethylglutaryl CoA (HMG-CoA) reductase (HMG-CoAR) [34]. When cellular cholesterol is increased, both cholesterol biosynthesis and lipoprotein uptake are reduced via feedback inhibition. In this condition, SREBP-2 activity is reduced to modulate gene expression, while the degradation of HMG-CoAR protein is enhanced in a post-translational manner [34].

Results from controlled feeding studies have been used to formulate predictive equations on the serum cholesterol response to dietary cholesterol [35]. These equations result in estimates ranging from 2.2–4.5 mg/dL changes in serum cholesterol per 100 mg/day change in dietary cholesterol [35]. More recent equations predict a 2.2–2.5 mg/dL change in serum cholesterol per 100 mg dietary cholesterol, equivalent to about a 2–3% change in serum cholesterol per egg [35]. This effect is relatively weak compared to modifying other dietary components, such as saturated fatty acids [36,37]. In fact, as early on as the 1960s, it was clear that dietary cholesterol was not a major factor in regulating serum cholesterol. Dr. Ancel Keys, a pioneer in studying diet-CVD relationships, stated in 1965, “For the purpose of controlling the serum level, dietary cholesterol should not be completely ignored but attention to this factor alone accomplishes little” [38]. Such small changes with dietary cholesterol intake are likely related to feedback control mechanisms that can limit our ability to absorb and synthesize cholesterol, as well as increase the amount that we excrete from our bodies [31,39]. Thus, most individuals have a marginal change in serum cholesterol in response to dietary cholesterol due to feedback regulation of whole body cholesterol stores. This is demonstrated by a rather extreme case, of an 88-year old man who apparently compulsively ate 20–30 eggs/day and had normal serum cholesterol (~200 mg/dL) [40]. This man was reported to absorb only a small fraction (18%) of the dietary cholesterol that he consumed and had twice the mean rate of bile acid synthesis as compared to control study volunteers [40]. Not everyone reacts the same way to dietary cholesterol intake, as the response is highly variable and depends on both genetic and metabolic factors [31,41,42]. Numerous clinical trials conducted in children [43], young women [44], men [45], and older adults [46] have demonstrated differences in serum cholesterol responses (hyper- vs hypo-responders) when consuming an additional 500–650 mg of dietary cholesterol from eggs for at least 4 weeks. The majority of the population (2/3) has no or only a mild increase in serum cholesterol when they consume a large amount of dietary cholesterol. These individuals are classified as hypo-responders or compensators, in that they can compensate by reducing cholesterol biosynthesis, absorption, and excretion [31,39]. On the other hand, a small proportion of the population has a much larger increase in serum cholesterol (≥2.3 mg/dL increase in serum cholesterol in response to 100 mg dietary cholesterol)—these individuals are classified as hyper-responders or non-compensators.

## 3. Dietary Cholesterol from Egg Intake and Lipoprotein Metabolism

### 3.1. Effects of Dietary Cholesterol from Egg Intake on LDL-C, HDL-C, and the LDL-C/HDL-C Ratio

Berger et al. [29] examined the serum lipid responses to dietary cholesterol across 19 intervention trials. Dietary cholesterol intake, which came mostly from eggs, was shown to significantly increase both serum LDL-C (6.7 mg/dL net change) and HDL-C (3.2 mg/dL net change), resulting in only a marginal increase in the LDL-C/HDL-C ratio (0.17 net change) [29]. Using the LDL-C/HDL-C ratio may provide an estimate of how much cholesterol is delivered to plaques via LDL, as well as potentially how much is being removed by HDL [47]. An LDL-C/HDL-C ratio <2.5 is considered optimal based on individual lipoprotein recommendations, while evidence suggests there is an increase in the risk for cardiovascular events above this level in some populations [47,48]. Table 1 summarizes results from clinical studies examining the effects of added dietary cholesterol via egg intake on serum lipids during weight maintenance in healthy and hyperlipidemic populations. In children and adults with normal cholesterol levels, consumption of 2–4 eggs per day vs. yolk-free egg substitute significantly increased both LDL-C and HDL-C in most studies, with no change in the LDL-C/HDL-C ratio [41,43,44,46]. Healthy men who were classified as hyper-responders (15 out of 40 participants) did show a significant increase in the LDL-C/HDL-C ratio with the consumption of three eggs per day for 30 days, however, the mean ratio (2.33 ± 0.80) was still within the optimal range of <2.5 [45]. Similar responses were observed in hyperlipidemic adults; consuming two eggs per day resulted in elevated HDL-C without a change in LDL-C in hypercholesterolemic adults, while there was an increase in both LDL-C and HDL-C in combined hyperlipidemics (elevated serum cholesterol and triglycerides) [49]. In older adults taking statins, consuming either two or four eggs per day did not significantly increase LDL-C, whereas HDL-C was increased with both doses of eggs [50].

Since there appears to be a relationship between dietary cholesterol and/or egg intake and heart disease in diabetics, do individuals with insulin resistance and/or diabetes have a more exaggerated lipid response? Table 2 summarizes results from clinical studies examining the effects of added dietary cholesterol via egg intake on serum lipids during weight maintenance in insulin-resistant and diabetic populations. Overall, those with insulin resistance and/or diabetes seem to have a weaker serum cholesterol response to eggs relative to leaner, insulin-sensitive individuals; consistent with the reduced dietary cholesterol absorption efficiency observed in obesity and metabolic syndrome [19,20,21]. Knopp et al. [41] compared the consumption of 4 eggs per day vs yolk-free egg substitute on serum lipids in both insulin-resistant lean individuals (IR) (mean BMI: 24.5 kg/m^2^) and insulin-resistant obese individuals (OIR) (mean BMI: 31.5 kg/m^2^). The 28-day consumption of eggs resulted in increases in both LDL-C and HDL-C in the IR group compared to egg substitute, while only HDL-C was significantly increased in the OIR group. Furthermore, clinical studies in diabetics showed that consuming 1–2 eggs per day for 5–6 weeks did not affect LDL-C or HDL-C relative to control groups lacking egg consumption [51,52].

Eggs are nutrient-dense, and relatively low in calories and carbohydrate, and thus, may be considered a good food choice for weight loss diets. Table 3 summarizes results from clinical studies examining the effects of added dietary cholesterol via egg intake on serum lipids during weight loss. Overall, the few weight loss studies conducted in overweight [53,54], insulin-resistant [55], and diabetic populations [56] have found no changes in LDL-C, with most increasing HDL-C. This led to most showing no effect on the LDL-C/HDL-C ratio [54,56], while one study showed a decrease in this ratio [55].

### 3.2. Effects of Dietary Cholesterol from Egg Intake on Lipoprotein Particle Characteristics

Although LDL-C and HDL-C are established indicators of CVD risk, lipoprotein particle characteristics, such as particle diameter and concentration, may also influence disease risk. For example, having a greater plasma concentration of particles of the large HDL subclass is strongly associated with lower risk for CVD, while the concentration of smaller HDL particles is less protective [57,58]. Conversely, the concentration of large LDL particles is only weakly associated with CVD risk, while small LDL concentrations are strongly positively linked with CVD [58]. The impact of smaller LDL particles on CVD is thought to be related to their greater susceptibility to oxidation compared with larger LDL particles [59]. Oxidized LDL is a major driver of atherosclerosis development and CVD [60]. Table 4 summarizes results from clinical studies examining the effects of added dietary cholesterol via egg intake on lipoprotein particle profiles during both weight maintenance and weight loss conditions. During weight maintenance, increases in LDL size and large LDL concentration are seen, sometimes at the expense of the more atherogenic small LDL [43,51,61,62]. Similar responses are observed in LDL particle profiles during weight loss [55,63]. The plasma concentration of oxidized LDL has been shown to be unaffected by added cholesterol from eggs in the few studies where it was measured [51,55,61]. Additionally, several studies examining the effects of added cholesterol from eggs under weight maintenance and weight loss conditions have shown increases in the size of HDL and the concentration of large HDL particles [55,62,63].

### 3.3. Effects of Dietary Cholesterol and/or Eggs on HDL Metabolism and Functionality

HDL is thought to be atheroprotective via its role in reverse cholesterol transport (RCT), as well as through antioxidant and anti-inflammatory activities [64]. Dietary cholesterol feeding in mice has been shown to result in a compensatory induction of RCT via HDL-related pathways [65]. Whether the reported increases in HDL-C and HDL particle size with dietary cholesterol and/or egg intake are improving RCT in humans is unclear. Multiple drug trials have failed to show a benefit of raising HDL-C in CVD [66,67]. However, there are studies that show dietary cholesterol and/or eggs may affect markers of HDL functionality beyond HDL-C. Dietary cholesterol intake in a cohort of 1400 adults was found to be one of only a few dietary factors to independently predict serum paraoxonase-1 (PON1) arylesterase activity [68]. PON1 is a lipolactonase enzyme carried by HDL known to promote atheroprotection through preventing lipoprotein oxidation [69], inhibiting macrophage inflammation [70], and enhancing HDL-mediated cellular cholesterol efflux [71]. Interestingly, serum PON1 arylesterase activity was significantly increased in young, healthy adults with the consumption of three eggs per day over 4 weeks [72]. Furthermore, increases in large HDL particle concentrations with egg intake have often coincided with increases in the activity of lecithin-cholesterol acyltransferase (LCAT) [45,46,54,55,72]. LCAT is an HDL-associated enzyme critical for the maturation of HDL via conversion of free cholesterol to cholesteryl esters [73]. The mobilization of cholesterol from macrophages by HDL, termed cholesterol efflux capacity, has been shown in cohort studies to be a significant predictor of CVD, independent of HDL-C and HDL particle concentrations [74,75]. Notably, when adults with metabolic syndrome consumed three eggs per day for 12 weeks during moderate carbohydrate restriction, the cholesterol efflux capacity of serum increased, whereas consumption of a yolk-free egg substitute had no effect [76]. Overall, the increased HDL-C commonly observed with dietary cholesterol feeding in humans appears to also coincide with improvements in other markers of HDL function. However, more investigation into the effects of dietary cholesterol on HDL functionality in humans is warranted.

### 3.4. The Phospholipid Component of Eggs May Influence the Response to Dietary Cholesterol

Relative to other foods, eggs are a rich source of phospholipids, such as phosphatidylcholine and sphingomyelin (SM) [27,77]. The phospholipid component of egg yolk may impair intestinal cholesterol absorption, which has been demonstrated with both phosphatidylcholine and SM [77]. Dietary SM, in particular, has been shown to inhibit the absorption of both cholesterol and fat in rodents [78,79]. The addition of SM to micelles inhibited cholesterol uptake in Caco-2 cells when it was used at increasing molar ratios of SM:cholesterol (≥0.5:1) [80]. The ratio of SM:cholesterol in egg yolk is approximately 1:2–1:4, and therefore, may modify the response to dietary cholesterol found in eggs. Dietary SM intake has been shown extensively in rodents, and less so in humans, to reduce serum lipids [81]. We recently fed egg SM (0.1% *w*/*w* diet) to male C57BL/6 mice that consumed a high fat, high cholesterol Western-type diet (60% kcal fat, 0.2% cholesterol by weight) for 10 weeks [82]. The egg SM content of the diet was fed at a similar ratio to dietary cholesterol as it is found in egg yolk. Compared to control mice fed the Western-type diet without SM, the mice fed egg SM had a 22% reduction in serum cholesterol, as well as 60% and 25% reductions in liver triglyceride and liver cholesterol, respectively. These mice fed egg SM were also protected from numerous inflammatory and metabolic complications associated with obesity. Recently, Chung et al. [83] conducted an extensive study on the effects of egg SM on atherosclerosis in apolipoprotein(apo) E^−/−^ mice fed both chow and high fat, high cholesterol diets. Chow-fed male apoE^−/−^ mice fed 1.2% (*w*/*w* diet) egg SM for 19 weeks were found to have reduced plaque size in aortic arches compared to control mice. Interestingly, serum TMAO concentrations were relatively unchanged even with this relatively high dose of egg SM, suggesting the phosphocholine moiety of egg SM is not readily available for trimethylamine generation by gut microbiota. Thus, it is likely that TMAO does not contribute to the effects of egg SM on disease outcomes in animal studies. The reduction in aortic arch lesion size with egg SM feeding was actually abolished when mice were co-administered broad-spectrum antibiotics to deplete gut microbiota. These findings suggest that dietary SM may work via effects on gut microbiota, which is supported by a recent mouse study showing dietary SM altered gut microbiota in high fat diet-fed mice [84]. More research is warranted to better understand the effects of dietary SM on chronic disease progression, including determining whether such effects are solely dependent on inhibiting lipid absorption or due to other reported effects on gut microbiota and inflammation [85].

## 4. Conclusions

**Figure 1:**
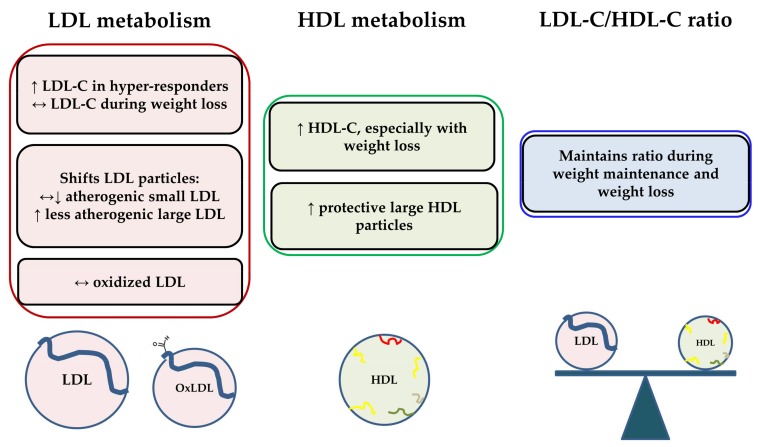
Summary of effects of consuming additional cholesterol from eggs on LDL and HDL metabolism in recent clinical studies. OxLDL = oxidized LDL; ↔ = no change relative to control, ↑ = increase relative to control; ↓ = decrease relative to control.

Figure 1 summarizes the effects of consuming additional cholesterol from eggs on LDL and HDL metabolism in recent clinical studies. Chronic daily egg intake does increase LDL-C to a certain extent in individuals classified as hyper-responders. However, LDL-C responses are typically minimal when eggs are consumed during weight loss conditions. Egg intake shifts LDL particles to the less detrimental, large LDL subclass, and does not appear to affect the levels of oxidized LDL. Egg intake also typically increases HDL-C and the concentration of large HDL, especially with weight loss. These changes appear to coincide with improvements in other markers of HDL function as well (e.g., PON1, cholesterol efflux capacity, LCAT). The effect of egg intake on the LDL-C/HDL-C ratio is negligible during weight maintenance and weight loss conditions. The relationship between dietary cholesterol and/or egg intake and CVD risk in diabetics requires further investigation. However, egg intake in the context of insulin resistance and/or diabetes would not be expected to be detrimental due to changes in serum lipids, as serum lipid responses to additional dietary cholesterol are often diminished in clinical studies of insulin-resistant groups compared to leaner, more insulin-sensitive individuals. Overall, recent intervention studies with eggs demonstrate that the additional dietary cholesterol does not negatively affect serum lipids, and in some cases, appears to improve lipoprotein particle profiles and HDL functionality.

## Figures and Tables

**Table 1:** Effects of additional dietary cholesterol from egg intake on serum lipids during weight maintenance: healthy and hyperlipidemic populations.

Study/Population	Design	# Days	LDL-C	HDL-C	LDL-C/HDL-C
Children					
Ballesteros et al. 2004 [43]; Healthy boys and girls	Crossover (*n* = 54): 2 eggs per day (518 mg cholesterol) vs. egg substitute	30	Hyper-: +25% Hypo-: ↔	Hyper-: +10% Hypo-: ↔	↔
Adults					
Herron et al. 2002 [44]; Healthy women	Crossover (*n* = 51): 3 eggs per day (640 mg cholesterol) vs. egg substitute	30	Hyper-: +20% Hypo-: ↔	Hyper-: +12% Hypo-: ↔	↔
Herron et al. 2003 [45]; Healthy men	Crossover (*n* = 40): 3 eggs per day (640 mg cholesterol) vs. egg substitute	30	Hyper-: +30% Hypo-: ↔	Hyper-: +8% Hypo-: ↔	Hyper-: + 22% Hypo-: ↔
Greene et al. 2005 [46]; Healthy older adults	Crossover (*n* = 42): 3 eggs per day (640 mg cholesterol) vs. egg substitute	30	Women: +10% Men: +2%	Women: +3% Men: +10%	↔
Knopp et al. 2003 [41]; Insulin-sensitive	Crossover (*n* = 65): 4 eggs per day (850 mg cholesterol) vs. egg substitute	28	+7%	+7%	ND *
Hyperlipidemic					
Knopp et al. 1997 [49]; Hypercholesterolemic (HC) and combined hyperlipidemic (CHL) men/women	Parallel: 2 eggs per day (425 mg cholesterol) (HC: *n* = 44; CHL: *n* = 31) vs. egg substitute (HC: *n* = 35; CHL: *n* = 21)	84	HC: ↔ CHL: +8% from baseline	HC: +8% from baseline CHL: +7% from baseline	ND
Vishwanathan et al. 2009 [50]; Statin-taking older adults	Crossover (*n* = 52): 2 or 4 eggs per day (~400–800 mg cholesterol) vs. egg exclusion	35	2 eggs: ↔ 4 eggs: ↔	2 eggs: +5% 4 eggs: +5%	ND

* ND, not determined. HDL-C = HDL cholesterol; Hyper- = hyper-responders; Hypo- = hypo-responders; LDL-C = LDL cholesterol; # = number of days of intervention; ↔ = no change relative to control.

**Table 2:** Effects of additional dietary cholesterol from egg intake on serum lipids during weight maintenance: insulin-resistant and diabetic populations.

Study/Population	Design	# Days	LDL-C	HDL-C	LDL-C/HDL-C
Insulin-resistant					
Knopp et al. 2003 [41]; Insulin-resistant (IR) and obese insulin-resistant (OIR)	Crossover (IR: *n* = 75; OIR: *n* = 57): 4 eggs per day (850 mg cholesterol) vs. egg substitute	28	IR: +6% OIR: ↔	IR: +6% OIR: +6%	ND *
Diabetic					
Ballesteros et al. 2015 [51]; Diabetic patients	Crossover (*n* = 29): 1 egg per day (250 mg cholesterol) vs. oatmeal breakfast	35	↔	↔	↔
Fuller et al. 2015 [52]; Diabetic patients	Parallel: High egg (12 eggs/week; ~300–350 mg cholesterol/day) (*n* = 72) vs. low egg (<2 eggs/week) (*n* = 68)	42	↔	↔	ND

* ND, not determined. HDL-C = HDL cholesterol; LDL-C = LDL cholesterol; # = *number of days of intervention*; ↔ = *no change* relative to control.

**Table 3:** Effects of additional dietary cholesterol from egg intake on serum lipids during weight loss.

Study/Population	Design	# Days	LDL-C	HDL-C	LDL-C/HDL-C
Harman et al. 2008 [53]; Men/women	Parallel: 2 eggs per day (~400 mg cholesterol) (*n* = 24) vs. egg exclusion (*n* = 21)	84	↔	↔	ND *
Mutungi et al. 2008 [54]; Overweight/obese men	Parallel: 3 eggs per day (640 mg cholesterol) (*n* = 15) vs. egg substitute (*n* = 13)	84	↔	+25% from baseline	↔
Pearce et al. 2011 [56]; Diabetic patients	Parallel: 2 eggs per day (590 mg cholesterol/day) (*n* = 31) vs. egg exclusion (213 mg cholesterol/day) (*n* = 34)	84	↔	Eggs +2% from baseline, egg exclusion −6% from baseline	↔
Blesso et al. 2013 [55]; Metabolic syndrome men/women	Parallel: 3 eggs per day (640 mg cholesterol) (*n* = 20) vs. egg substitute (*n* = 17)	84	↔	+17% from baseline	↓

* ND, not determined. HDL-C = HDL cholesterol; LDL-C = LDL cholesterol; # = number of days of intervention; ↔ = no change relative to control; ↓ = decrease relative to control.

**Table 4:** Effects of additional dietary cholesterol from egg intake on lipoprotein particle profiles.

Study/Population	Design	# Days	LDL Particles	Oxidized LDL	HDL Particles
Weight maintenance					
Ballesteros et al. 2004 [43]; Healthy children	Crossover (*n* = 54): 2 eggs per day (518 mg cholesterol) vs. egg substitute	30	↑Large LDL (+31% LDL-1 in hyper-) ↓Small LDL (−38% LDL-3 in hyper-) ↑LDL size	ND *	ND
Herron et al. 2004 [61]; Healthy men/women	Crossover (*n* = 52): 3 eggs per day (640 mg cholesterol) vs. egg substitute	30	↑Large LDL (+13% LDL-1, +30% LDL-2 in women hyper-)	↔	ND
Greene et al. 2006 [62]; Healthy elderly men/women	Crossover (*n* = 42): 3 eggs per day (640 mg cholesterol) vs. egg substitute	30	↑Large LDL (+30% from baseline in hyper-)	ND	↑Large HDL (+23% from baseline in hyper-) ↑HDL size
Ballesteros et al. 2015 [51]; Diabetic patients	Crossover (*n* = 29): 1 egg per day (250 mg cholesterol) vs. oatmeal breakfast	35	↔	↔	↔
Weight loss					
Mutungi et al. 2010 [63]; Overweight/obese men	Parallel: 3 eggs per day (640 mg cholesterol) (*n* = 15) vs. egg substitute (*n* = 13)	84	↑Large LDL (+42% from baseline)	ND	↑Large HDL (+52% from baseline) ↑HDL size
Blesso et al. 2013 [55]; Metabolic syndrome men/women	Parallel: 3 eggs per day (640 mg cholesterol) (*n* = 20) vs. egg substitute (*n* = 17)	84	↑Large LDL (+22% from baseline)	↔	↑Large HDL (+30% from baseline) ↑HDL size

* ND, not determined. Hyper- = hyper-responders; # = number of days of intervention; ↔ = no change relative to control, ↑ = increase relative to control; ↓ = decrease relative to control.
